# The alkaloids of *Banisteriopsis caapi*, the plant source of the Amazonian hallucinogen Ayahuasca, stimulate adult neurogenesis *in vitro*

**DOI:** 10.1038/s41598-017-05407-9

**Published:** 2017-07-13

**Authors:** Jose A. Morales-García, Mario de la Fuente Revenga, Sandra Alonso-Gil, María Isabel Rodríguez-Franco, Amanda Feilding, Ana Perez-Castillo, Jordi Riba

**Affiliations:** 1 Instituto de Investigaciones Biomédicas (CSIC-UAM), Arturo Duperier 4, 28029 Madrid, Spain; 2 Center for Networked Biomedical Research on Neurodegenerative Diseases (CIBERNED), 28031 Madrid, Spain; 3 Departamento de Biología Celular, Facultad de Medicina, UCM, Plaza Ramón y Cajal s/n, 28040 Madrid, Spain; 4 Human Neuropsychopharmacology Research Group. Sant Pau Institute of Biomedical Research (IIB-Sant Pau). Sant Antoni María Claret, 167. 08025 Barcelona, Spain; 5 Instituto de Química Médica (IQM-CSIC), Juan de la Cierva 3, 28006 Madrid, Spain; 6 The Beckley Foundation, Beckley Park, Oxford, OX3 9SY United Kingdom; 7 Centro de Investigación Biomédica en Red de Salud Mental, CIBERSAM, Planta 028029 Madrid, Spain; 8 MFR currently at: Department of Physiology and Biophysics, Virginia Commonwealth University School of Medicine, Richmond, VA 23298 USA

## Abstract

*Banisteriopsis caapi* is the basic ingredient of ayahuasca, a psychotropic plant tea used in the Amazon for ritual and medicinal purposes, and by interested individuals worldwide. Animal studies and recent clinical research suggests that *B*. *caapi* preparations show antidepressant activity, a therapeutic effect that has been linked to hippocampal neurogenesis. Here we report that harmine, tetrahydroharmine and harmaline, the three main alkaloids present in *B*. *caapi*, and the harmine metabolite harmol, stimulate adult neurogenesis *in vitro*. In neurospheres prepared from progenitor cells obtained from the subventricular and the subgranular zones of adult mice brains, all compounds stimulated neural stem cell proliferation, migration, and differentiation into adult neurons. These findings suggest that modulation of brain plasticity could be a major contribution to the antidepressant effects of ayahuasca. They also expand the potential application of *B*. *caapi* alkaloids to other brain disorders that may benefit from stimulation of endogenous neural precursor niches.

## Introduction

Ayahuasca is the Quechua name used to designate *Banisteriopsis caapi*, a jungle liana of the Malpighiaceae family that is native to the Amazon and Orinoco river basins^1^. The term is also applied to the tea that is obtained by infusing in water the stems of *B*. *caapi*, alone or in combination with other plants^2^. The ayahuasca tea is a central element in the ancient shamanic practices and rites of passage of the indigenous inhabitants of northwestern South America. More recently, ayahuasca has become a central sacrament in the rituals of Brazilian syncretic religious groups. These so-called “ayahuasca religions” have popularized ayahuasca use with the expansion of their activities to North America and Europe^3^.

Ayahuasca has a complex chemistry and pharmacology. *B*. *caapi* contains high amounts of harmine and tetrahydroharmine (THH) and to a lesser degree harmaline, three indole alkaloids with β-carboline structure^4^. These alkaloids are reversible monoamine-oxidase-A (MAO-A) inhibitors^5^, while THH can also inhibit serotonin reuptake^6^. Although *B*. *caapi* can be the sole ingredient of the tea^7^, up to 100 different plants have been described as admixtures to ayahuasca. These plants contain a wide variety of psychotropic substances such as nicotine (from *Nicotiana* spp.), scopolamine (from *Brugmansia* spp.), caffeine (from *Ilex guayusa and Paullinia yoco*), cocaine (from *Erythoxylum coca*) and *N*,*N*-dimethyltryptamine (DMT, from *Psychotria viridis* and *Diplopterys cabrerana*)^2, 8^.

In recent years, non-traditional use of ayahuasca has expanded worldwide, especially the version of the tea that combines *B*. *caapi* with *P*. *viridis*, due to the visionary effects induced by DMT, a psychedelic serotonin-2A agonist^9^. This expansion and reports of health benefits derived from its use have stimulated research into the pharmacology and therapeutic potential of ayahuasca^10^. Two recent studies found that a single dose of ayahuasca rapidly reduced depressive symptoms in treatment-resistant patients^11, 12^. Remarkably, clinical improvement was maintained for up to three weeks. With the resurgence of research on psychedelics^13^, the β-carbolines of *B*. *caapi* have been considered to play a minor role in the overall pharmacology of ayahuasca preparations. However, this is probably a narrow view. *B*. *caapi* is the common ingredient to all ayahuasca brews, and chemical analyses have shown that while the β-carbolines are present in all ayahuasca samples, this is not always the case for DMT^14, 15^. For instance, an ayahuasca sample from the Brazilian Church *União do Vegetal*, a group known to regularly combine *B*. *caapi with P*. *viridis* in their ayahuasca, was found to contain no DMT at all^16^.

Due to their ubiquitous presence in ayahuasca, it can be hypothesized that the β-carbolines contribute to the CNS effects of the tea. Studies in animals have shown that harmine has antidepressant effects in behavioral animal models of depression^17, 18^. Responses after harmine in the forced swim and open field tests are analogous to those obtained with ayahuasca infusions prepared from *B*. *caapi* and *P*. *viridis* and containing DMT^19, 20^. These findings suggest that DMT is not essential for the behavioral responses observed in animals. Additionally, in contrast with more traditional antidepressants such as imipramine, harmine increases BDNF levels in the hippocampus after both acute and chronic administration^18, 21^. These data suggest that harmine and potentially the other β-carbolines present in *B*. *caapi* contribute to the therapeutic effects of ayahuasca observed in clinical studies involving patients with depression^11, 12^.

At the cellular level, antidepressant drug action has been linked to the ability of drugs to stimulate adult neurogenesis^22^. Neurogenesis is the process of generating functional neurons from progenitor cells. In the adult brain of mammals, neurogenesis occurs in two main niches: the subventricular zone (SVZ) of the lateral ventricle and the subgranular zone of the dentate gyrus of the hippocampus (SGZ). Neural stem cells in these areas can be induced to asymmetrically divide, generating new stem cells, and astrocytes, oligodendrocytes or neurons^23, 24^. These newly generated neurons have the capacity to migrate and integrate into existing neural circuits. The activity and phenotypic fate of neural stem cells is determined by both endogenous and exogenous factors^25^. Beyond understanding the mechanisms of adult neurogenesis, we are ultimately interested in its therapeutic capacity. Specifically, despite their various mechanism of action, clinically-effective antidepressants share the common feature of inducing neural stem cell proliferation and differentiation into new neurons^26–28^.

Here, we investigated the capacity of the three main β-carbolines present in *B*. *caapi* to induce neurogenesis *in vitro* using neural progenitor cells from adult mice. We decided to focus on the alkaloids found in all ayahuasca brews, rather than on nicotine, DMT, scopolamine or other psychoactive compounds that can be present in ayahuasca following the addition of the admixture plants mentioned above. To this end neurospheres were prepared from stem cells obtained from both the SVZ and the SGZ and treated with harmine, THH, harmaline, and harmol. The latter is present only in small amounts in *B*. *caapi* but is readily formed *in vivo* in humans via O-demethylation of harmine, which undergoes extensive first-pass metabolism^4, 29, 30^. As shown below, results showed that the *B*. *caapi* β-carboline alkaloids present in ayahuasca directly regulate proliferation, migration and differentiation of neural stem cells.

## Results

### *B*. *caapi* β-carbolines control the activity of neural progenitors

**Figure 1:**
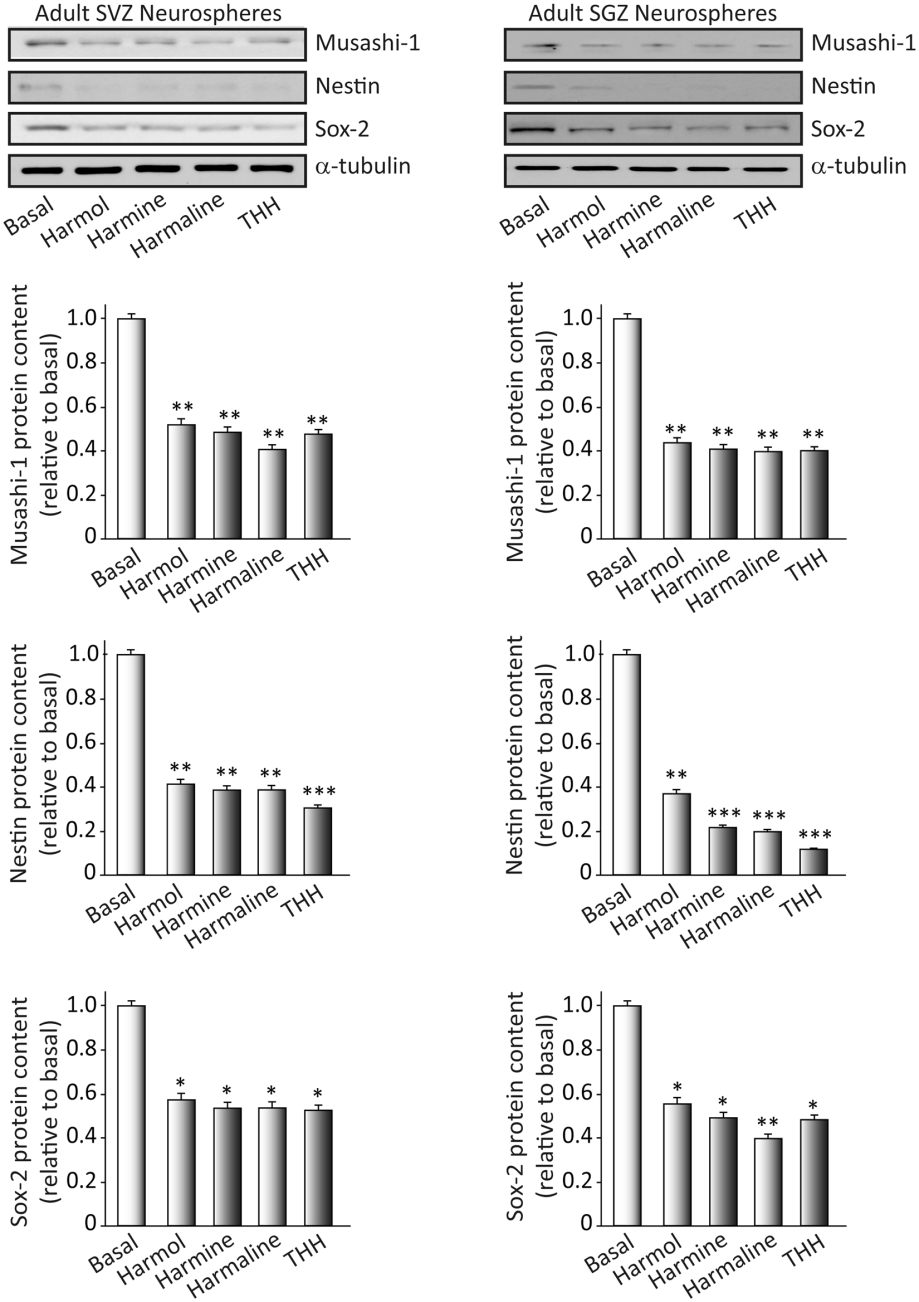
Effects of ayahuasca β-carboline alkaloids on stemness of cultured adult neurospheres. Representative Western blots and bar graphs showing expression levels of the precursor cell markers musashi-1, nestin and SOX-2 after treatment with each of the four alkaloids tested (1 µM). Values in bar graphs indicate mean ± SD of the quantification of at least three independent experiments corresponding to four different cellular pools. The left side of the image shows results for the subventricular zone (SVZ) of the brain. The right side of the image shows results for the subgranular zone of the hippocampus (SGZ). *p ≤ 0.05; **P ≤ 0.01; ***p ≤ 0.001 indicate significant results in the post-hoc pair-wise comparisons (Bonferroni) versus non-treated (basal) cultures.

We isolated neural stem cells from the SVZ and the SGZ and we cultured them as free-floating neurospheres. Neurospheres from adult tissue are characterized by self-renewal and multipotent differentiation. To study the “stemness” of cultured neurospheres, we analyzed the expression of the following proteins: (a) musashi-1, a marker of undifferentiation; (b) nestin, an intermediate filament protein characteristic of neural stem/progenitor cells; and (c) “sex determining region Y-box 2” (SOX-2), a transcription factor essential for maintaining self-renewal and pluripotency of undifferentiated stem cells. We treated the neurosphere cultures for 7 days under proliferative conditions with each of the four β-carbolines: harmol, harmine, harmaline and tetrahydroharmine, all at 1 µM concentration. After treatment, we isolated the proteins and performed Western blots. Figure 1 clearly shows significant reductions in the amount of musashi-1, nestin and SOX-2 in the SVZ (musashi: F(4,15) = 49.87, p < 0.001; nestin: F(4,15) = 50.105, p < 0.001; SOX-2: F(4,15) = 121.684, p < 0.001) and SGZ (musashi: F(4,15) = 32.819, p < 0.001; nestin: F(4,15) = 141.556, p < 0.001; SOX-2: F(4,15) = 22.572, p < 0.001) neurospheres following treatment with the test compounds. These results indicated that the β-carbolines promote a loss of “stemness” or undifferentiated state of the neurospheres.

### *B*. *caapi* β-carbolines induce proliferation and growth in neurosphere cultures

**Figure 2:**
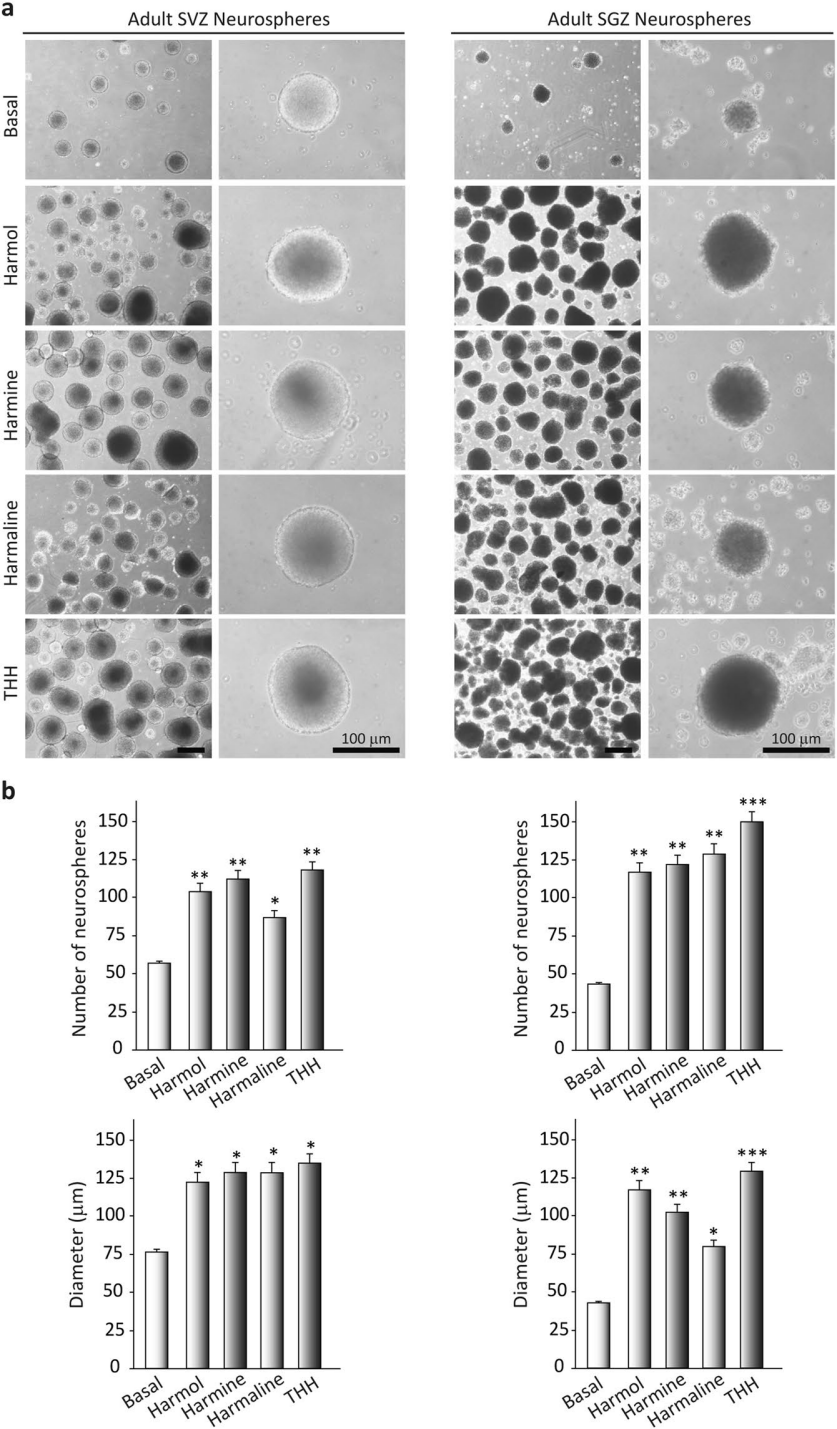
Effects of ayahuasca β-carboline alkaloids on adult neurosphere formation. (**a**) Representative phase-contrast micrographs showing the number and size of neurospheres after 7 days in culture in the presence of each of the four alkaloids tested (1 µM). The number and diameter of at least 50 neurospheres was determined in control and treated cultures. Scale bar = 100 μm. (**b**) Bar graphs showing results as mean values ± SD of the quantification of at least three independent experiments corresponding to four different cellular pools. The left side of the image shows results for the subventricular zone (SVZ) of the brain. The right side of the image shows results for he subgranular zone of the hippocampus (SGZ). *p ≤ 0.05; **p ≤ 0.01; ***p ≤ 0.001 indicate significant results in the post-hoc pair-wise comparisons (Bonferroni) versus non-treated (basal) cultures.

We analyzed next whether the treatment with test compounds modulated proliferation capacity in the neurosphere. For this purpose, we grew free floating neurospheres under proliferative conditions, i.e., in the presence of epidermal growth factor (EFG) and fibroblast growth factor (FGF) for 7 days, with the addition of saline (control) or each of the four tested β-carbolines. As shown in Fig. 2, addition of the alkaloids to the cultures markedly increased the rate of formation and the size of the neurospheres.

**Figure 3:**
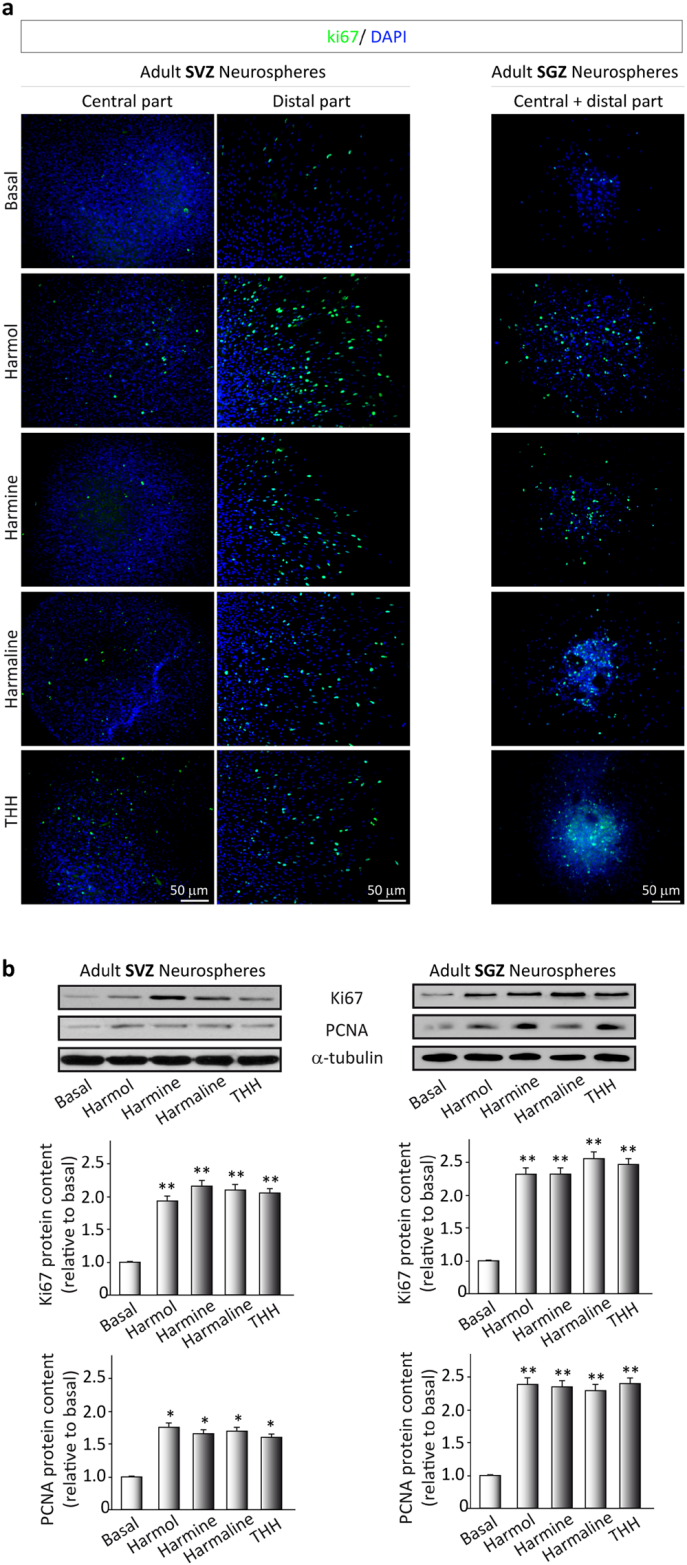
Effects of ayahuasca β-carboline alkaloids on adult neural stem cells proliferation. (**a**) Representative immunofluorescence images showing the expression of the cellular marker for proliferation ki67 (green) in neurospheres derived from the subventricular zone (SVZ) of the lateral ventricle and the subgranular zone (SGZ) of the hippocampus. SVZ-derived neurospheres are shown in two panels showing the central part of the sphere (left) and the distal migration site (right). Single images from SGZ-derived neurospheres show the whole neurosphere, including the central and distal areas. DAPI was used for nuclear staining. Scale bar = 50 μm. (**b**) Representative Western blots of ki67 and the proliferating cell nuclear antigen (PCNA) levels in neurospheres treated for 7 days with each of the four alkaloids tested (1 µM). Bar graphs show the results of the quantification analyses. Each bar indicates relative protein levels expressed as mean ± SD of the quantification of at least three independent experiments corresponding to four different cellular pools. The left side of the image shows results for the subventricular zone (SVZ) of the brain. The right side of the image shows results for he subgranular zone of the hippocampus (SGZ). *p ≤ 0.05; **p ≤ 0.01 indicate significant results in the post-hoc pair-wise comparisons (Bonferroni) versus non-treated (basal) cultures.

After 7 days of β-carboline treatment, the number of SVZ-derived neurospheres was significantly higher than in the vehicle-treated cultures (F(4,15) = 466.512, p < 0.001). Analogous results were observed for neurospheres derived from the SGZ (F(4,15) = 1226.445, p < 0.001). Regarding the size of the neurospheres, significant increases were observed for both SVZ- and SGZ-derived neurospheres after β-carboline treatment, as compared with non-treated cultures (SVZ: F(4,15) = 126.858, p < 0.001); SGZ: F(4,15) = 172.617, p < 0.001). In view of these results, we next analyzed the expression of Ki67, a marker of dividing cells, and “proliferating cell nuclear antigen” (PCNA) (Fig. 3).

As expected, treatment of the cultures with the β-carbolines resulted in an increase in the number of Ki67- (SVZ: F(4,15) = 514.405, p < 0.001); SGZ: F(4,15) = 51.976, p < 0.001) and PCNA-positive cells (SVZ: F(4,15) = 7.905, p < 0.001); SGZ: F(4,15) = 42.213, p < 0.001), indicating a direct effect of these compounds on proliferation. These results indicated that the β-carbolines actively stimulate the proliferation and growth of neurospheres from both the SVZ and the SGZ by controlling the activity of neural progenitors.

### *B*. *caapi* β-carbolines increase neural stem cell migration

In addition to proliferation and differentiation, neurogenesis involves the migration of neural stem cells and their integration into functional circuits. We thus tested next the effects of the β-carbolines on precursor migration. We treated the neurospheres with each of the test compounds and we monitored migration of the newly formed cells using livescanning microscopy for 48–72 h. The results are presented in Supplementary Figure 1 (and in the Supporting Information Videos 1–5). As shown therein, β-carboline treatment resulted in significant increases in migration capacity (SVZ: F(4,15) = 349.872, p < 0.001); SGZ: F(4,15) = 1203.666, p < 0.001) in comparison with basal (control). Neural stem cells moved long distances out of the neurosphere body in the presence of the four tested compounds. On the contrary, cells in the control cultures remained close to the neurosphere core.

### *B*. *caapi* β-carbolines induce differentiation of neural stem cells

**Figure 4:**
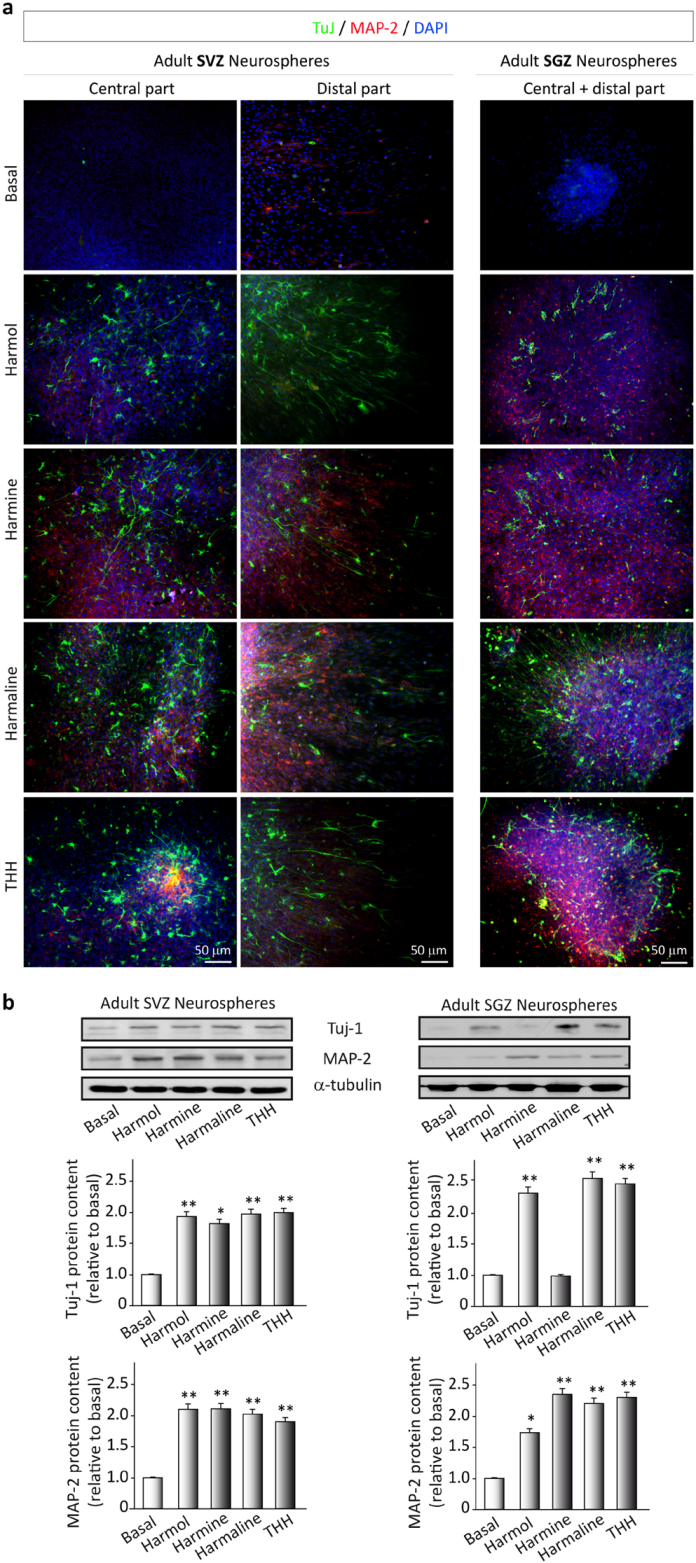
Ayahuasca β-carboline alkaloids promote stem cell differentiation towards a neuronal phenotype. After 7 days on culture in the presence of harmol, harmine, harmaline and tetrahydroharmine (THH), free floating neurospheres derived from the adult subgranular (SGZ) and subventricular (SVZ) zone were adhered on coated coverslips and allowed to differentiate for 3 days in the presence of alkaloids at 1 µM. (**a**) Representative immunofluorescence images showing the expression of the neuronal markers β-III-Tubulin (TuJ-1 clone, green) and MAP-2 (red) in neurospheres. DAPI was used for nuclear staining. SVZ-derived neurospheres are shown in two panels showing the central part of the sphere (left) and the distal migration site (right). Single images from SGZ-derived neurospheres show the whole neurosphere, including the central and distal areas. Scale bar = 50 μm. (**b**) Representative Western blots of β-tubulin and MAP-2. Quantification analyses are also shown. Results are the mean ± SD of the quantification of at least three independent experiments corresponding to four different cellular pools. The left side of the image shows results for the subventricular zone (SVZ) of the brain. The right side of the image shows results for he subgranular zone of the hippocampus (SGZ). *p ≤ 0.05; **p ≤ 0.01 indicate significant results in the post-hoc pair-wise comparisons (Bonferroni) versus non-treated (basal) cultures.

We investigated the capacity of the β-carbolines to promote cell differentiation into any of the three different cellular types that form the central nervous system: astrocytes, neurons and/or oligondendrocytes. We analyzed the formation of the different cell types using immunocytochemistry. Neurosphere cultures prepared from the SVZ and the SGZ were adhered to a substrate and cultured under differentiation conditions, that is, in the absence of growth factors and in the presence of 1% fetal bovine serum. The β-carbolines were added to the medium and, after 3 days in culture, we evaluated the expression of Tuj1- and MAP-2-positive cells by immunocytochemistry analysis (Fig. 4a) and we quantified these proteins by Western blot (Fig. 4b). After the three-day period, vehicle-treated cultures only showed a few positively-stained cells for Tuj-1 or MAP-2. On the contrary, in the β-carboline-treated cultures, the number of both Tuj1- (SVZ: F(4,15) = 16.295, p < 0.001); SGZ: F(4,15) = 45.997, p < 0.001) and MAP-2-positive cells (SVZ: F(4,15) = 41.631, p < 0.001); SGZ: F(4,15) = 22.951, p < 0.001) was significantly increased. These results indicate that the test compounds induce the differentiation of neural stem cells towards mature neurons.

**Figure 5:**
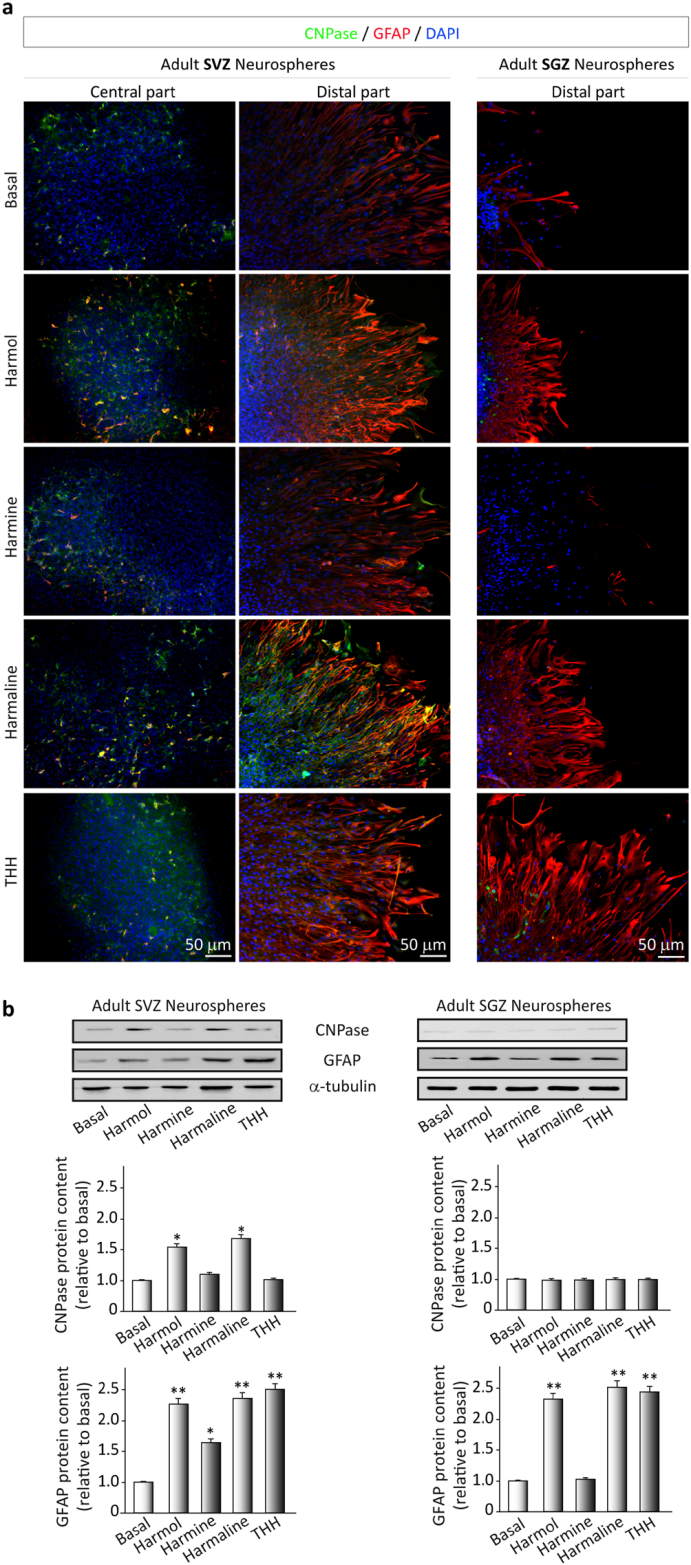
Ayahuasca β-carboline alkaloids promote astrogliogenesis. Neurospheres derived from the adult subgranular (SGZ) and subventricular (SVZ) zone were cultured in the presence of harmol, harmine, harmaline and tetrahydroharmine (THH). After 7 days neurospheres were adhered on coated coverslips and allowed to differentiate for 3 days in the presence of alkaloids at 1 µM. (**a**) Neurosphere immunofluorescence images showing in green the expression of CNPase (oligodendrocyte marker) and glial fibrillary acidic protein (GFAP, red) that stains astrocytes. SVZ-derived neurospheres are shown in two panels showing the central part of the neurosphere (left) and the distal migration site (right). Single images from SGZ-derived neurospheres show the distal part of the neurosphere. DAPI was used for nuclear staining. Scale bar = 50 μm. (**b**) Representative Western blots of CNPase and GFAP. Quantification analyses are also shown. Results are the mean ± SD of the quantification of at least three independent experiments corresponding to four different cellular pools. The left side of the image shows results for the subventricular zone (SVZ) of the brain. The right side of the image shows results for he subgranular zone of the hippocampus (SGZ). *p ≤ 0.05; **p ≤ 0.01 indicate significant results in the post-hoc pair-wise comparisons (Bonferroni) versus non-treated (basal) cultures.

Concerning the formation of astrocytes or oligodendrocytes, Fig. 5 shows that most of the test compounds did not induce the differentiation of neural precursors towards an oligondedrocyte phenotype. Results showed that only the harmol- and harmaline-treated SVZ cultures presented some scattered CNPase-positive cells (Fig. 5a). These results were further confirmed by quantification of the Western blots (Fig. 5b; SVZ: F(4,15) = 7.853, p < 0.001); SGZ: F(4,15) = 1.472, p > 0.05)). In the case of GFAP-positive cells, a high number was observed in basal conditions (Fig. 5) and this number was increased after β-carboline treatment (SVZ: F(4,15) = 43.838, p < 0.001); SGZ: F(4,15) = 136.268, p < 0.001), suggesting that these compounds also promote the differentiation of astroglial cells or astrocyte-like radial cells.

## Discussion

Here we showed that adult neural stem cell activity is regulated by harmine, THH, and harmaline, the most abundant alkaloids in *B*. *caapi* and ayahuasca, and by harmol, the main metabolite of harmine in humans^31^. Using an *in vitro* model of adult neurogenesis, we found that all four β-carbolines stimulated the proliferation and migration of progenitor cells and promoted their differentiation predominantly into neurons.

The four compounds tested effectively promoted proliferation, migration, and differentiation of progenitor cells obtained from the SVZ and the SGZ, the two main niches of adult neurogenesis in rodents. The β-carbolines increased the number and size of primary neurospheres, induced the loss of the neurospheres’ undifferentiated state, and promoted subsequent cell migration and differentiation mainly into a neuronal phenotype, as indicated by the positive expression of β-III-tubulin and MAP2, but also into astroglial cells. Taken together, these three effects indicate that *B*. *caapi* alkaloids have the capacity to regulate the expansion and fate of stem cell populations.

Analysis of the proliferation stage showed that all four β-carbolines increased the number and size of neurospheres, the number of Ki-67-stained cells, and the amounts of Ki-67 and PCNA protein as measured by Western blot. Our results for the effect of harmine on proliferation are in line with a previous study showing harmine-induced increase in mitosis in cultured chick embryo cells^32^, and in human neural progenitor cells^33^. To our knowledge, the effects of harmaline and THH on neurogenesis have not been studied before. While harmaline is only present in small amounts in *B*. *caapi*, THH is the second most abundant β-carboline in the plant^4, 29^. Additionally, THH shows more consistent plasma levels between individuals and studies than harmine, which is rapidly degraded to harmol when taken orally^29, 34^. The latter, formed *in vivo* by O-demethylation of the parent compound^29^, showed proliferative effects of similar magnitude to those of harmine.

Our results showed that *B*. *caapi* β-carbolines promoted cellular migration and differentiation, suggesting that these alkaloids not only act as mitogens for neural stem cells, but also modulate cellular fate. The largest effects on migration were observed for harmaline and THH. Increased migration capacity is relevant in certain conditions such as brain injury, where stem cell niches are far from the damaged area^35–37^. All tested compounds also promoted cellular differentiation. Neural stem cells are known to differentiate into neurons, astrocytes, and oligodendrocytes^23, 24^. The observed increases in Tuj-1 and MAP-2 protein expression indicated differentiation predominantly toward a neuronal phenotype. In the SVZ both proteins were equally expressed after each of the four treatments. However, in the SGZ harmine administration did not influence Tuj-1 levels, a marker of immature neurons, but significantly increased the expression of MAP-2, suggesting a larger impact on neuronal maturation.

All the above indicate that *B*. *caapi* β-carbolines facilitate neurogenesis at multiple levels. This capacity is of interest, since in pathological conditions the replacement of neurons may be optimized by acting simultaneously on various processes^38, 39^. The effect of the β-carbolines on cellular proliferation and differentiation is not unique to these compounds, having been observed for endogenous molecules such as leukotriene B4^40^, BMPs^41^, the growth factors EGF/FGF2^42^, and NGF/BDNF/bFGF^43^, and the transcription factors Lmx1a and Lmx1b^44^. However, the fact that the β-carbolines also stimulated migration highlights the versatility of these exogenous compounds, as they can promote the three processes involved in full adult neurogenesis.

A likely possible explanation for the observed effects of β-carbolines in neurogenesis is the increase in monoamine levels caused by MAO inhibition. With this said, we must acknowledge that the magnitude of the neurogenic effects was similar for the four compounds, despite harmol and THH being inhibitors that are between a hundred and a thousand times weaker than harmine or harmaline^5^. Moreover, the role of monoamines in neurogenesis is not fully understood. Knocking out the 5-HT_1A_ receptor in mice impaired neurogenesis after fluoxetine but not after imipramine, indicating that neurogenesis was independent from elevated serotonin levels^45^. In another study, the authors reported the unexpected finding that serotonin depletion actually promoted hippocampal neurogenesis instead of decreasing it^46^. In a recent paper, harmine, but not the MAO inhibitor pargyline, stimulated proliferation of human neural progenitor cells *in vitro* ^33^. Harmine effects were mediated through inhibition of the DYRK1A kinase rather than through MAO inhibition. This opens the possibility that the β-carbolines tested here regulated stem cell fate via DYRK1A or other alternative mechanisms. To our knowledge, the inhibitory effects of harmaline, tetrahydroharmine and harmol on DYRK1A has not been examined. Other potential molecular targets for the neurogenic effects of small molecules include the modulation of the GSK-3β/β-catenin pathway ^47^, upregulation of the brain-derived neurotrophic factor^48^, increased levels of vascular endothelial growth factor^49^; and glucocorticoid receptor activation^50^. Future studies should assess whether *B*. *caapi* β-carbolines interact with one or more of these pathways.

Our findings have relevant therapeutic implications. The association between neurogenesis and anti-depressant activity is well documented^45, 51^. Enhanced hippocampal neurogenesis reduces depression-like behaviors in animals^51^. Furthermore, clinically effective antidepressants stimulate neurogenesis, independent of their chemical structure and mechanism of action. To cite a few examples, chronic treatment with the serotonin reuptake inhibitor fluoxetine increases neurogenesis in rats^52, 53^, as does chronic treatment with the selective MAO-A inhibitor pirlindole^53^. The association between neurogenesis and antidepressant effect is not limited to rodents and pharmacological interventions. Electroconvulsive therapy in primates also stimulates proliferation of neural precursors in the hippocampus and their differentiation into neurons^54^. Hippocampal neurogenesis appears to be necessary for antidepressant action. Irradiation of the SGZ of the hippocampus in mice prevents the neurogenic and behavioral effects of fluoxetine and imipramine^45^.

In humans, two recent clinical studies have demonstrated rapid and long-lasting antidepressant effects after a single ayahuasca dose in patients who did not respond to conventional treatment^11, 12^. The therapeutic potential of ayahuasca is an area of increasing research interest beyond depression^55^. Alterations in adult neurogenic niches have been associated with a number of pathologies affecting the central nervous system^56–59^. Stimulation of these niches is currently being investigated as a novel therapeutic strategy for neuropsychiatric disorders^60–62^. Regular ayahuasca use has been associated decreases in problematic alcohol, cocaine and opiate consumption, indicating anti-addiction properties for *B*. *caapi* preparations^63, 64^. These potential anti-addictive properties are particularly relevant if we acknowledge the notorious difficulty of treating substance use disorders. Drug-dependent patients not only show functional deficits in reward processing and cognitive control, but also structural alterations in brain gray and white matter^65^.

Our study has a series of limitations that need to be acknowledged. Ayahuasca brews contain other active compounds that were not tested here. A popular version of ayahuasca in the USA and Europe contains DMT, a serotonergic psychedelic^9^. It is possible that DMT may have contributed to the antidepressant effects reported in clinical studies using ayahuasca^11, 12^. This contribution could be due to both brain plasticity mediated by 5-HT_2A_ receptor activation^66^ and to the profound psychological experiences induced by psychedelics^67^. While studying DMT in the neurogenesis model was not an objective of the present investigation, it could be assessed in a future study, comparing it with other 5-HT_2A_ agonists such as psilocybin or LSD. Although several animal studies have already shown that harmine improves behavioral measures of depression^17, 18^, future studies could ideally test the four compounds assessed here for both *in vivo* neurogenesis and behavioral improvement. Finally, future research could also use positive controls to compare the potency of the *B*. *caapi* β-carbolines with that of other antidepressants, such as SSRIs and MAO inhibitors.

In conclusion, here we showed that the β-carboline alkaloids present in *B*. *caapi*, the plant source of the ayahuasca tea, promote neurogenesis *in vitro* by stimulating neural progenitor pool expansion, and by inducing cellular migration and differentiation into a neuronal phenotype. The stimulation of neurogenic niches in the adult brain may substantially contribute to the antidepressant effects reported for ayahuasca in recent clinical studies. The versatility and full neurogenic capacity of the *B*. *caapi* β-carbolines warrant further investigation of these compounds. Their ability to modulate brain plasticity indicates their therapeutic potential for a broad range of psychiatric and neurologic disorders.

## Methods

### Ethics

Animals used in this study were cared for following the animal experimental procedures previously approved by the “Ethics Committee for Animal Experimentation” of the Instituto de Investigaciones Biomédicas and carried out in accordance with the European Communities Council, directive 2010/63/EEC and National regulations, normative 53/2013.

### Drugs

Harmine and harmaline were obtained from commercial sources (Sigma-Aldrich). Harmol and tetrahydroharmine were obtained by synthesis following the procedures described below.

### Synthesis of harmol and tetrahydroharmine

#### 1-Methyl-9*H*-pyrido[3,4-*b*]indol-7-ol hydrobromide (harmol·HBr)

To a solution of harmine (151 mg, 0.71 mmol, Sigma-Aldrich) in acetic acid (3 mL) hydrobromic acid (3 mL, 47% aqueous solution) was added. The mixture was heated at 160 °C during 25 min in a microwave oven and then allowed to cool to room temperature. After few minutes at 25 °C, needles started to form and harmol·HBr·H_2_O (180 mg, 85% yield) was recovered by filtration as a yellow solid of mp 250–251 °C. ^1^H NMR (400 MHz, D_2_O) δ 7.62 (d, *J* = 5.4 Hz, 1 H), 7.39 (d, *J* = 5.4 Hz, 1 H), 7.18 (d, *J* = 8.4 Hz, 1 H), 6.26 (d, *J* = 8.5 Hz, 1 H), 5.96 (s, 1 H), 2.31 (s, 3 H). ^13^C NMR (101 MHz, D_2_O) δ 158.55, 143.69, 134.75, 132.07, 131.27, 127.49, 122.97, 112.87, 112.18, 111.48, 95.12, 14.77. HPLC purity 100% (230 to 400 nm). HRMS (ESI^+^): *m/z* calcd for C_12_H_10_N_2_O: 198.07931, found: 198.07934. Anal. Calcd for C_12_H_10_N_2_O·HBr·H_2_O (297.15): C, 48.50; H, 4.41; N, 9.43. Found: C 48.08; H 4.29; N 9.52.

#### 7-Methoxy-1-methyl-2,3,4,9-tetrahydro-1*H*-pyrido[3,4-*b*]indole hydrochloride (tetrahy-droharmine·HCl, THH·HCl)

Sodium borohydride (680 mg, 18.00 mmol) was added in portions to a stirred solution of harmaline (1.54 g, 7.20 mmol, Sigma-Aldrich) in water (40 mL) at 0 °C and the mixture was acidified until pH 2 with aqueous HCl (2 M). After 75 min at room temperature, the mixture was made alkaline with NaOH (10% aqueous solution), extracted with dichloromethane (3 × 20 mL) and evaporated to dryness. Solid was dissolved in isopropyl alcohol, heated and then treated with an excess of concentrated HCl until a precipitate appeared. After 3 days at 4 °C, THH·HCl was collected by filtration (1.40 g, 77% yield) as a white solid of mp 201–202 °C. ^1^H NMR (400 MHz, DMSO) δ 10.98 (s, 1 H), 7.34 (d, *J* = 8.6 Hz, 1 H), 6.85 (d, *J* = 2.2 Hz, 1 H), 6.68 (dd, *J* = 8.6, 2.2 Hz, 1 H), 4.68 (q, *J* = 6.8 Hz, 1 H), 3.76 (s, 3 H), 3.59 (dt, *J* = 12.4, 4.7 Hz, 1 H), 3.37–3.27 (m, 1 H), 2.97–2.81 (m, 2 H), 1.59 (d, *J* = 6.7 Hz, 3 H). ^13^C NMR (101 MHz, DMSO) δ 155.93, 136.95, 129.62, 120.17, 118.75, 108.97, 105.14, 94.67, 55.19, 48.42, 40.40, 18.30, 17.24. HPLC purity 98% (230 to 400 nm). Anal. Calcd for C_13_H_16_N_2_O·HCl (252.74): C, 61.78; H, 6.78; N, 11.08. Found: C 61.46; H 6.86; N 10.84.

### Adult precursor isolation

Neural stem cells (NSCs) were isolated from the subgranular zone (SGZ) of the hippocampus and the subventricular zone (SVZ) of the lateral ventricle of adult C57BL/6 mice (3 months of age) and prepared following previously described methods^68^. A total number of 40 animals were used throughout the whole study, divided in four pools of 10 animals each. Every pool was individually used to perform all the experiments described in this section. Briefly, tissue was carefully dissected, dissociated in DMEM medium with glutamine, gentamicin and fungizone and then digested with 0.1% trypsin-EDTA + 0.1% DNAase + 0.01% hialuronidase for 15 min at 37 °C. The isolated stem cells were seeded into 12-well dishes at a density of ~40,000 cells per cm^2^ in DMEM/F12 (1:1) containing 10 ng/mL epidermal growth factor (EGF), 10 ng/mL fibroblast growth factor (FGF) and N2 medium.

### Neurosphere cultures and treatments

NSCs were cultured under standard conditions and in the presence of growth factors (EGF and FGF) for a week, in 12-well dishes. After this time, small neural progenitor-enriched growing spheres known as neurospheres (NS) were formed. At this point, with all NS having the same stage and size, cultures were treated daily for 7 days with vehicle or 1 μM solutions of each of the four β-carbolines tested: harmol, harmine, harmaline and tetrahydroharmine (THH). The effective dose of compounds was chosen based on our previous studies on β-carboline analogs^69^ and the 7-day period was selected as a standard time frame used previously by our own and other research groups to test the neurogenic potential of drugs^37, 50, 68, 70^. The 1 μM concentrations chosen for the β-carbolines was lower than the 7.5 μM found to induce optimal proliferation in a previous *in vitro* study with harmine^33^ but closer to the plasma concentrations of 0.52–1.52 μM reported for harmine and THH, respectively, in a recent study involving ayahuasca administration to healthy humans^71^. At these concentrations, none of the tested β-carbolines affected the viability of the cultured cells. Proliferation and growth analysis was assessed from these cultures and the number and diameter of NS were quantified using the Nikon Digital Sight, SD-L1 software. All the NS contained in ten wells per condition were counted. Some of these proliferating NS were used for immunoblotting analysis, while others were seeded onto poly-L-lysine precoated-6-well plates, and/or -coverslips and cultured in the presence of β-carboline alkaloids (1 μM) under proliferation conditions (medium containing 1% fetal bovine serum and without exogenous growth factors). Once neurospheres were differentiated (72 h), those grown on coated 6-well plates were used for immunoblotting and those on coverslips for immunocytochemical analysis. The remaining NS were cultured for 48–72 h under differentiation conditions onto μ-Slide 8-well plates (Ibidi, Martinsried, Germany) and used for migration assays.

### Immunocytochemistry

Cells were processed for immunocytochemistry as previously described^69^. Briefly, NS grown on glass were previously fixed 15 minutes at room temperature in 4% paraformaldehyde and incubated at 37 °C for 1 h with primary antibodies directed against ki67 (1/200, rabbit, Abcam), β-III-tubulin (1/400, TuJ-1 clone; rabbit; Abcam), MAP-2 (1/200, mouse; Sigma), CNPase (rabbit, Covance) and GFAP (1/500, mouse; Sigma). After several rinses in PBS, samples were then incubated with Alexa-488 goat anti-rabbit and Alexa-647 goat anti-mouse antibodies (1/500, Molecular Probes) for 45 min at 37 °C. Staining of nuclei was performed using 4′,6-diamidino-2-phenylindole (DAPI, 1/500). Finally images were acquired in a LSM710 laser scanning spectral confocal microscope (Zeiss). Confocal microscope settings were adjusted to produce the optimum signal-to-noise ratio.

### Immunoblot analysis

Differentiated cultured NS were re-suspended in ice-cold cell lysis buffer (Cell Signaling Technology) with protease inhibitor cocktail (Roche) and incubated for 15–30 min on ice. A total amount of 30 µg of protein was loaded on a 10% or 12% SDS-PAGE gel and transferred to nitrocellulose membranes (Protran, Whatman). The membranes were blocked in Tris-buffered saline with 0.05% Tween-20 and 5% skimmed milk or 4% BSA (MAP-2 blots), incubated with primary and secondary antibodies, and washed according to standard procedures. Primary antibodies used were: musashi-1 (1/500, rabbit; Abcam), nestin (1/500, rabbit; Abcam), SOX-2 (1/1000, mouse, Cell Signaling), ki67 (1/500, rabbit, Abcam), PCNA (1/500, mouse, Millipore), β-III-tubulin (1/1000, TuJ clone; mouse; Covance), MAP-2 (1/500, mouse; Sigma), CNPase (1/500, rabbit, Covance), GFAP (1/1000, mouse; Sigma) and α-tubulin (1/5000, mouse; Sigma). Secondary peroxidase-conjugated (1/1000) donkey anti-rabbit (Amersham Biosciences, GE Healthcare), or rabbit anti-mouse antibodies (Jackson Immunoresearch) were used. Values in figures are the average of the quantification of at least three independent experiments each of them corresponding to four different cellular pools.

### Cellular migration assay

Free-floating single spheres were individually picked up with a pipette and plated onto μ-Slide 8-well plates as previously described^70^. Images were acquired with a Cell Observer system from Zeiss (Jena, Germany) using a Zeiss Observer Z1 microscope, equipped with a Cascade 1 K camera, a monitorized X/Y stage, and a Module S incubator with equipment for temperature and CO_2_ control. During all experiments cells were kept in a humidified atmosphere of 5% CO2 in air at 37 °C. Axio Vision Rel. 4.8 software (Zeiss) for time-lapse imaging and cell tracking was used. Phase contrast images of cells were taken every 60 min using a × 4 objective (Achromatic, Zeiss). Cell migration was scored in at least 10 independent experiments per condition. The farthest distance of cell migration was calculated from the edge of the sphere. Resulting movies were collected and exported as avi files and are shown at 5 frames per second (See Supporting Videos 1–5).

### Statistical analysis

Data were analyzed using a one-way ANOVA with treatment as factor (control, harmol, harmine, harmaline, THH). Significant results in the ANOVA (*p* < *0*.*05*) were followed by post-hoc pair-wise test (Bonferroni). The SPSS statistical software package (version 20.0) for Windows (Chicago, IL, USA) was used for all statistical analyses.

## Electronic supplementary material

Supplementary Information

Supplementary_Video_1_Basal

Supplementary_Video_2_Harmol

Supplementary_Video_3_Harmine

Supplementary_Video_4_Harmaline

Supplementary_Video_5_Tetrahydroharmine
