# Supplementary material for: The alkaloids of Banisteriopsis caapi, the plant source of the Amazonian hallucinogen Ayahuasca, stimulate adult neurogenesis in vitro
Source: Sci Rep. 2017 Jul 13;7:5309. doi: 10.1038/s41598-017-05407-9 (PMC5509699; doi:10.1038/s41598-017-05407-9)
Supplement: Supplementary file 1 — Supplementary Information [file 41598_2017_5407_MOESM1_ESM.doc]

**Supplementary Information**

**Supplementary Figure 1 Legend.** **Ayahuasca** **β-carboline alkaloids promote adult neural stem cell migration.** (A) Effects of each alkaloid on cell migration out of the neurospheres. Single neurospheres were plated onto μ-Slide 8-well plates in the presence each compound and cell migration out of the sphere was monitored and quantified 24 h later. Representative photomicrographs are shown. Scale bars = 50 μm. (B) Quantitative data indicating farthest distance of cell migration. The left side of the image shows results for the subventricular zone (SVZ) of the brain. The right side of the image shows results for he subgranular zone of the hippocampus (SGZ). *p ≤ 0.05; **p ≤ 0.01; ***p ≤ 0.001 indicate significant results in the post-hoc pair-wise comparisons versus non-treated (basal) cultures after Bonferroni correction for multiple comparisons.

**Supplementary Figure 1**


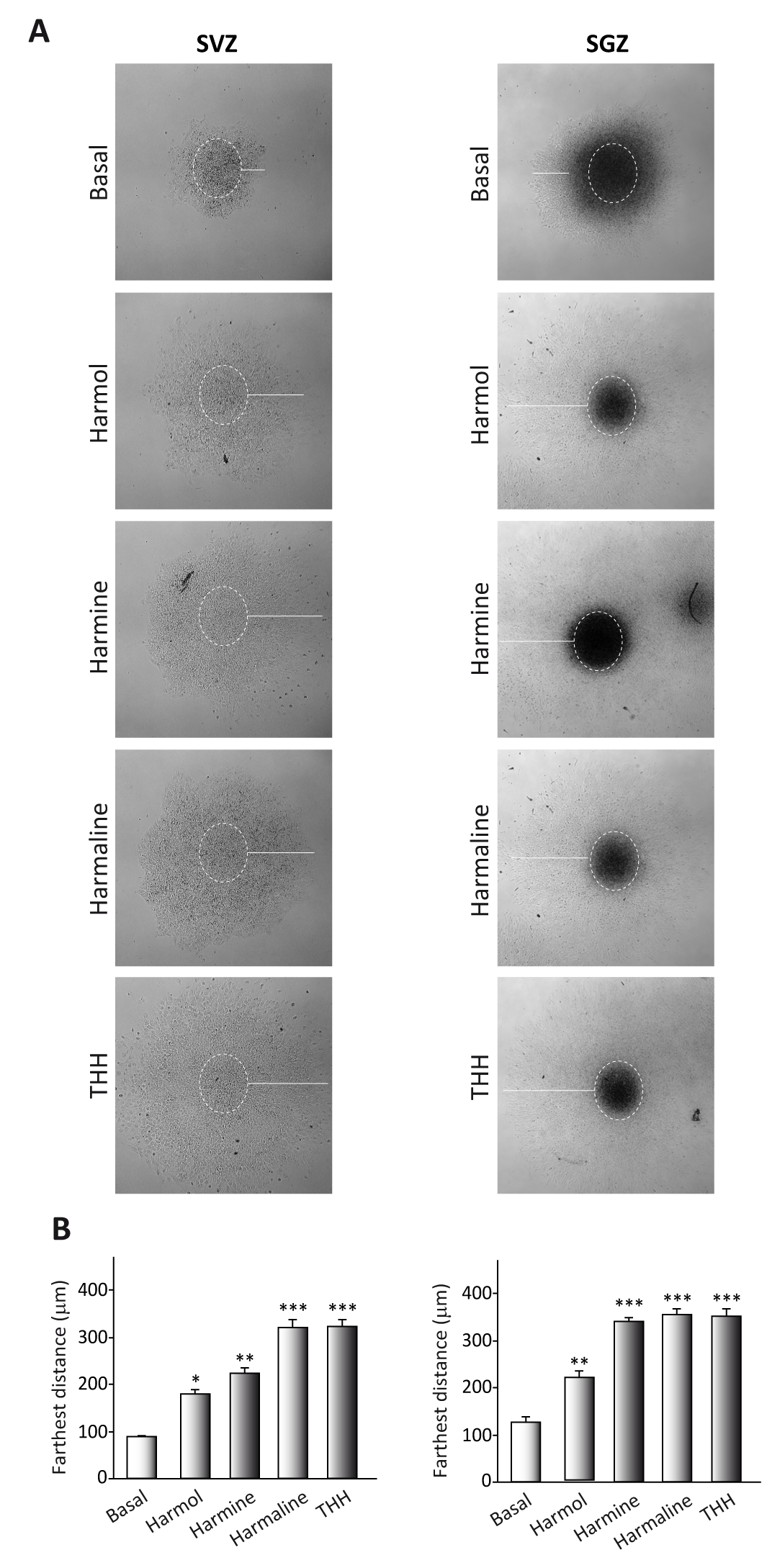


**Legends of the Supporting Video files**

**Supporting Information Video 1 (Basal)**

Video recording of cell migration pattern from basal (saline-treated) neurospheres. Single spheres were plated and migration monitored for 48-72h, taking phase contrast images every 60 min.

**Supporting Information Video 2 (Harmol)**

Video recording of cell migration pattern from harmol-treated neurospheres. Single spheres were plated and migration monitored for 48-72h, taking phase contrast images every 60 min.

**Supporting Information Video 3 (Harmine)**

Video recording of cell migration pattern from harmine-treated neurospheres. Single spheres were plated and migration monitored for 48-72h, taking phase contrast images every 60 min.

**Supporting Information Video 4 (Harmaline)**

Video recording of cell migration pattern from harmaline-treated neurospheres. Single spheres were plated and migration monitored for 48-72h, taking phase contrast images every 60 min.

**Supporting Information Video 5 (Tetrahydroharmine)**

Video recording of cell migration pattern from tetrahydroharmine (THH)-treated neurospheres. Single spheres were plated and migration monitored for 48-72h, taking phase contrast images every 60 min.
